# Effect of waste cooking oil addition on ammonia emissions during the composting of dairy cattle manure

**DOI:** 10.5713/ab.21.0343

**Published:** 2022-01-03

**Authors:** Kazutaka Kuroda, Akihiro Tanaka, Kenichi Furuhashi, Naoki Fukuju

**Affiliations:** 1 Division of Livestock Research, Kyushu Okinawa Agricultural Research Center, National Agriculture and Food Research Organization, Koshi 861-1192, Japan; 2 Department of Biological and Environmental Engineering, Graduate School of Agricultural and Life Sciences, The University of Tokyo, Tokyo 113-8657, Japan

**Keywords:** Aeration Condition, Ammonia Emissions, Composting, Dairy Cattle Manure, Waste Cooking Oil

## Abstract

**Objective:**

The objective of this study was to evaluate the effect of waste cooking oil (WCO) addition on ammonia (NH_3_) emissions during the composting of dairy cattle manure under two aeration conditions.

**Methods:**

The composting tests were conducted using the laboratory-scale composting apparatuses (14 L of inner volume). Three composting treatments (Control, WCO1.5, and WCO3, with WCO added at 0 wt%, 1.5 wt%, and 3 wt% of manure, respectively) were performed in two composting tests: aeration rate during composting was changed from 0.55 to 0.45 L/min in Test 1, and fixed at 0.3 L/min in Test 2, respectively. The NH_3_ emitted and nitrogen losses during the composting were analyzed, and the effect of the addition of WCO on NH_3_ emissions were evaluated.

**Results:**

Both tests indicated that the composting mixture temperature increased while the weight and water content decreased with increasing WCO content of the composting mixtures. On the other hand, the NH_3_ emissions and nitrogen loss trends observed during composting in Tests 1 and 2 were different from each other. In Test 1, NH_3_ emissions and nitrogen losses during composting increased with increasing WCO contents of the composting samples. Conversely, in Test 2, they decreased as the WCO contents of the samples increased.

**Conclusion:**

The WCO addition showed different effect on NH_3_ emissions during composting under two aeration conditions: the increase in WCO addition ratio increased the emissions under the higher aeration rate in Test 1, and it decreased the emissions under the lower aeration rate in Test 2. To obtain reduction of NH_3_ emissions by adding WCO with the addition ratio ≤3 wt% of the manure, aeration should be considered.

## INTRODUCTION

Composting is the process of preparing compost from organic material, typically organic waste, for use as plant fertilizer. In Japan, composting is commonly used to recycle livestock manure [1–3]. During composting, microorganisms actively decompose and stabilize the organic matter in manure. The heat generated by decomposition dries the manure and kills pathogens, weed seeds, and parasite eggs. However, composting also emits environmentally harmful gases, such as malodorous compounds and greenhouse gases [4,5]. Among these, ammonia (NH_3_) is emitted in large quantities, thereby increasing odor-related complaints from the communities around the composting facilities, and increasing the occurrence of global environmental pollution events, such as acid rain and soil acidification [6,7]. Additionally, nitrogen loss via NH_3_ emissions decreases the value of compost for use as a fertilizer. Therefore, reducing NH_3_ emissions is essential to ensure efficient composting of livestock manure.

Substantial NH_3_ emissions during composting are typically attributed to a high ratio of nitrogen to biodegradable carbon sources in composting materials. Finstein and Morris [8] reported that NH_3_ was emitted during the composting of municipal solid waste with a carbon to nitrogen ratio (C/N ratio) <25. However, the C/N ratio of animal manure is typically <20, which indicates that composting of animal manure will produce more NH_3_ [9–11]. If additional biodegradable carbon sources are added to the composting material, the growth of microorganisms, and the assimilation of nitrogen by these microorganisms will be promoted, thereby reducing NH_3_ emissions. Therefore, many studies have examined the effects of adding carbon sources during the composting of nitrogen-rich materials, including animal manure, on NH_3_ emissions [11].

In recent years, several studies have reported lower NH_3_ emissions were observed after the addition of cooking oil (CO) or waste cooking oil (WCO) during composting of animal manure [12–14] and rabbit food (as a model of organic waste) [15]. The lipids in CO and WCO are easily degradable carbon sources for microorganisms, and these studies suggested that NH_3_ emissions from composting can be reduced by adding CO and WCO. However, this method has not been widely used in composting of animal manure in the practical composting facilities.

In this study, laboratory-scale composting tests of dairy cattle manure were conducted to obtain basic information on the use of adding WCO to reduce NH_3_ emissions. Additionally, the effect of aeration on the application of this method was assessed.

## MATERIALS AND METHODS

### Materials for composting

Dairy cattle manure was collected from a dairy farmer in Kikuchi, Kumamoto Pref., Japan, and transported to our research center 1 d prior to each composting test. A small amount of straw, used as bedding material in cattle stalls, was contaminated in the manure, was removed before the composting. Sawdust was purchased from a composting center (Koshi Bio X, Koshi, Japan) and WCO was obtained from Hayashi Sangyo Co. Ltd., Kumamoto, Japan.

### Composting tests

#### Composting apparatus

**Figure 1:**
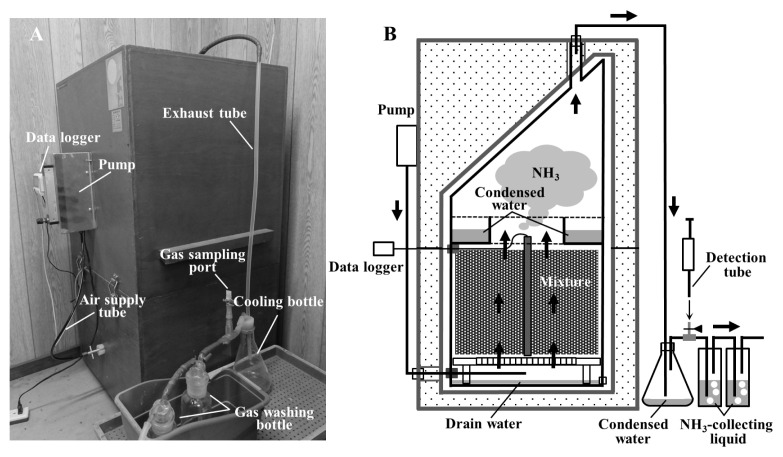
Composting apparatus: (A) photograph; (B) schematic view in use. Arrows indicate the flow of air.

The composting tests were conducted using laboratory-scale composting apparatus (Kaguyahime, Fujihira Kogyo Co. Ltd., Tokyo, Japan), as described in Kuroda et al [16] (Figure 1A). The main part of the apparatus was a stainless cylinder vessel with an effective capacity of approximately 14 L, which was filled with composting mixture. Further details are given in Table 1. A thermocouple sensor rod, connected to a data logger (TR-7wf; T&D Corporation, Matsumoto, Japan) was inserted into the mixture to record the temperature fluctuations during composting. The vessel was placed in an insulation case, and the mixture was aerated at specific flow rates, as described in Table 1, from the bottom of the cylinder by an air pump. A cooling bottle, gas sampling port, and two gas-washing bottles with NH_3_-collecting liquid (6 N H_2_SO_4_ solution) were connected to the exhaust tube.

#### Composting test conditions

Table 1 shows the parameters set for the composting tests. The mixtures used for composting were prepared with dairy cattle manure, sawdust, and WCO. Two composting tests (Tests 1 and 2) with different aeration conditions were conducted. In both tests, three treatments were used: Control, WCO1.5, and WCO3 with WCO added to the dairy cattle manure and sawdust mixture at weights of 0%, 1.5%, and 3% of the manure, respectively; additionally, the composting period was set to 20 d. In Test 1, the aeration rate was set at 0.55 L/min for the first 7 d of composting, corresponding to ≈37 L/min/m^3^ of the initial composting material, and was later decreased to 0.50 L/min (7 to 14 d) and 0.45 L/min (14 to 20 d). In Test 2, the aeration rate was set at 0.30 L/min throughout the composting process, corresponding to ≈23.4 L/min/m^3^ of the initial composting material. This aeration rate was substantially lower than that of large-scale composting treatments, which are typically 50 to 300 L/min/m^3^ of the composting material, and usually ≥100 L/min/m^3^ of the material [17]. Both tests were conducted in triplicates under the same parameters.

During composting, the NH_3_ concentration in the exhaust gas was measured using a detection tube (No. 3L or 3M; Gastec Co., Ayase, Japan) at the gas sampling port at intervals of 12 or 24 h. Water condensed outside the apparatus was retained in the cooling bottle and collected after every NH_3_ concentration measurement. The condensed water in the vessel was stored in the reservoir placed under the cap, and the drain water eluting from the mixture was retained at the bottom of the vessel (Figure 1B). On days 7 and 14, the mixture was removed from the apparatus, mixed completely, and returned to the apparatus (turning). In Test 1, the aeration rate was changed after turning. Some amounts of the mixture were collected at the beginning and end of the composting. At the turnings and the end of the composting, the NH_3_-collecting liquid, condensed water, and drain water retained in the vessel were collected and used for further analyses.

### Sample analyses

The mixtures and liquids collected during the composting tests were analyzed according to the methods described in Kuroda et al [16,18]. The water content, volatile solids (VS, an indicator of organic matter), and ash in the mixture were analyzed by drying the mixture at 105°C for 1 d, followed by combustion at 550°C for 6 h. For pH measurement, an extract of the mixture was prepared using a 2 N KCl solution.

Ammonium nitrogen (NH_4_-N) and nitrous or nitric nitrogen (NO_x_-N) in the mixture were analyzed based on the methods of Bremner and Keeney [19], using a 2 N KCl extract solution. Kjeldahl-nitrogen (Kj-N) in the mixture was analyzed according to the methods described in Kuroda et al [20] based on the method of Bremner [21]. Further, organic nitrogen and total nitrogen in the mixture were determined by subtracting NH_4_-N from Kj-N and adding Kj-N and NO_x_-N, respectively. The NH_3_-collecting liquid, condensed water, and drain water were mixed, and the dissolved NH_4_-N content was analyzed from this liquid mixture. The NH_4_-N content in the liquid mixture was determined as collected NH_4_-N during composting. The water content, VS, pH, NH_4_-N, and NO_x_-N analyses were duplicated, and the Kj-N analysis was quintuplicated for each sample. Based on these analyses, the total nitrogen composition of the initial and final mixtures and the nitrogen losses that occurred during composting were calculated and subjected to Tukey’s test for assessing statistical significance.

## RESULTS

### Composting test (Test 1)

**Figure 2:**
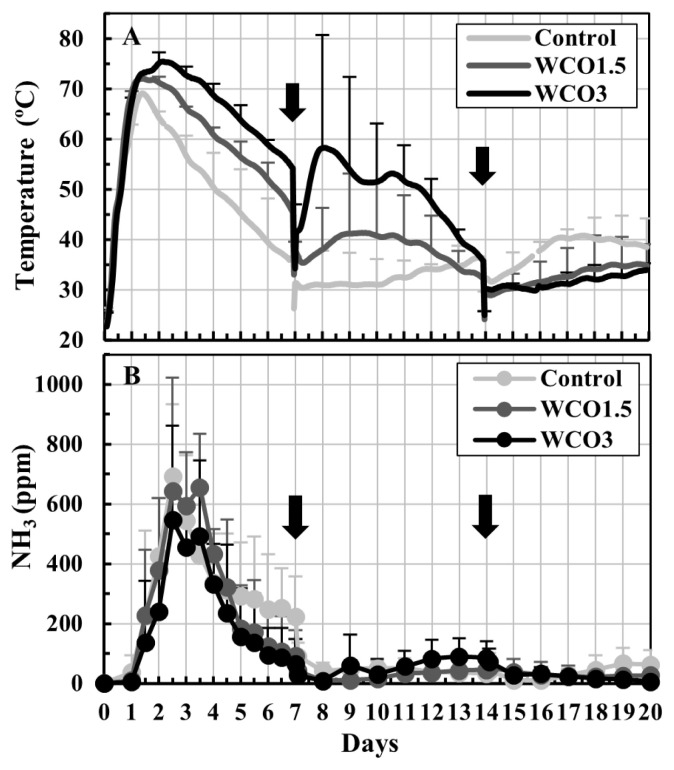
Changes in temperatures of the mixtures and NH_3_ concentrations in the exhaust gases during Test 1. The means of the measured values at each measurement time point during repeated composting tests are plotted on the graphs. The bars on the lines indicate standard deviations (n = 3). The black ink density of the bar correspond to the respective treatments. The arrows indicate turning.

Changes in the temperatures of the composted mixtures and the NH_3_ concentrations in the exhaust gases during Test 1 are shown in Figure 2. During the first 14 d of composting, high temperatures were recorded for the mixtures with high WCO contents (Figure 2A). After the second turning on day 14, the temperatures of all three mixtures remained below 41°C. The NH_3_ concentrations increased considerably within the first 7 d of composting, and peaked within 2 to 4 d. The peak NH_3_ mean concentration values were 693, 655, and 547 ppm for Control, WCO1.5, and WCO3 treatments, respectively. After the first turning, the NH_3_ concentrations of all three mixtures remained below 100 ppm (Figure 2B).

Table 2 shows the composition of the initial and final mixtures, the losses that occurred during composting, and pH values for Test 1. The pH of the initial mixtures decreased with an increase in the WCO content, and the pH increased by the end of the test, reaching 8.3 to 8.7. At the beginning of composting, the VS content of the mixtures increased proportionally with the WCO content across the three treatments, whereas the water and ash contents were similar for all treatments. During composting, the total weights, water contents, and VS of the three mixtures decreased. The weights and water contents of the final mixtures decreased and the weight loss during composting increased with an increase in the WCO content. Significant differences (p<0.05) were observed between the total weights of the final mixtures in Control and WCO3 treatments, and between the water contents of the final mixtures and weight losses of all three treatments. However, composting did not affect the ash content in any of the treatments. The condensed water and drain water collected during composting accounted for 65% to 70% of the weight losses in the treatments. Moreover, the amount of condensed water increased with an increase in the WCO content, and significant differences (p<0.05) were observed between the condensed water amounts in Control treatment and those in WCO1.5 and WCO3.

The total nitrogen and the respective forms of nitrogen in the initial mixtures were similar for all samples. During composting, NH_4_-N and total nitrogen in the mixtures decreased and the nitrogen losses increased in Control treatment, followed by WCO1.5 and WCO3. Compared with the initial mixtures, the organic nitrogen content in the final mixtures increased marginally in all three treatments. Further, less than 0.2 g of NO_x_-N was detected in the three mixtures at the beginning and end of the tests. The collected NH_4_-N accounted for 88% to 90% of nitrogen losses in the treatments, and a significant difference was observed between the nitrogen losses except the collected NH_4_-N (“Other loss” in Table 2) in Control and WCO3 treatments. The average ratios of nitrogen losses to the total nitrogen contents in the initial mixtures were 15.2%, 19.2%, and 21.7% in Control, WCO1.5, and WCO3 treatments, respectively. These ratios were approximately 26% higher in WCO1.5 and 43% higher in WCO3 than in Control.

### Composting test (Test 2)

**Figure 3:**
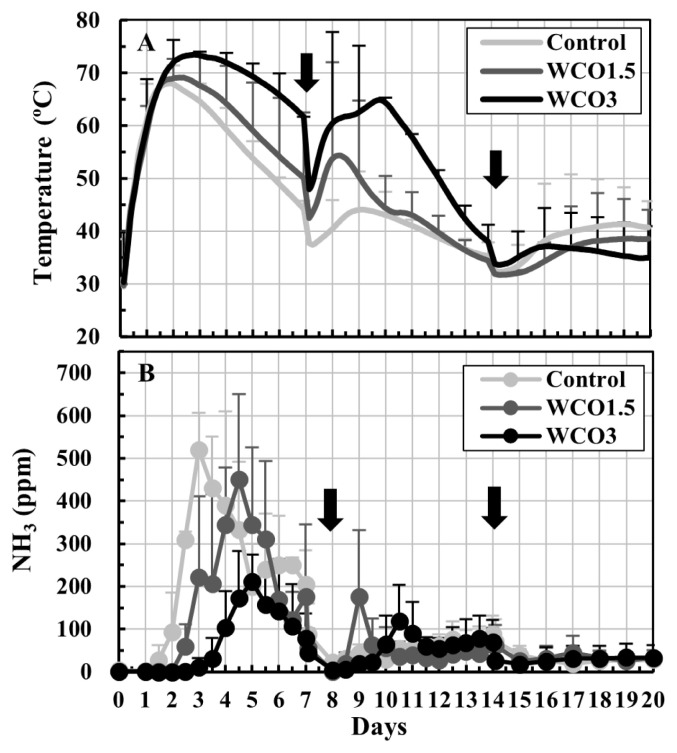
Changes in temperatures of the mixtures and NH_3_ concentrations in the exhaust gases during Test 2. The means of the measured values at each measurement time point during repeated composting tests are plotted on the graphs. The bars on the lines indicate standard deviations (n = 3). The black ink density of the bar correspond to the respective treatments. The arrows indicate turnings.

The trends of changes in temperatures of the composted mixtures and NH_3_ concentrations in the exhaust gases observed in Test 2 were similar to those in Test 1 (Figure 3). During the first 14 d of the test, high temperatures were recorded in the samples having high WCO contents (Figure 3A). Considerable increases in the NH_3_ concentrations were observed in the first 7 d, and the concentrations peaked 3 to 5 d from the beginning of the test and occurred slightly later than in Test 1. The peak NH_3_ mean concentrations for Control, WCO1.5, and WCO3 treatments were 520, 450, and 212 ppm, respectively (Figure 3B).

Changes in the pH and the primary composition of the mixtures during composting were similar to those in Test 1 (Table 3). The initial pH, which were low in the mixtures having a high WCO content, increased to 8.6 to 8.7 during composting. In the initial mixtures, the water and ash contents were the same for all samples, and the VS contents increased proportionally with the WCO content. Moreover, the total weights and water contents of the final mixtures decreased, and significant weight losses were observed, with increasing WCO contents. Significant differences (p<0.05) were observed between the weights of Control and WCO3 samples, and between the water contents and weight losses of samples of all three treatments. However, the ash contents in the treatments remained unaffected by composting. The condensed water and drain water accounted for 68% to 78% of the weight losses during composting in the three treatments. Moreover, the condensed water increased with increasing WCO content, with significant differences (p<0.05) observed between those in Control and those in WCO1.5 and WCO3.

The total nitrogen and the respective forms of nitrogen in the initial mixtures were similar for all samples. Furthermore, increases in the WCO content of the mixtures decreased the nitrogen loss and NH_4_-N content of the mixtures during composting. By the end of the composting, the organic nitrogen content of the mixtures marginally increased, while NO_x_-N contents (≤0.07 g) remained unchanged in all three treatments, which was similar to the observations of Test 1. The collected NH_4_-N accounted for 89% to 91% of the nitrogen losses in the treatments. Moreover, significant differences (p<0.05) were observed in the nitrogen losses and the collected NH_4_-N of the WCO3 and Control treatments. The average ratios of the nitrogen losses to the total nitrogen contents in the initial mixtures were 12.3%, 11.0%, and 9.4% in Control, WCO1.5, and WCO3 treatments, respectively, and these ratios were approximately 11% and 24% lower in WCO1.5 and WCO3 treatments, respectively, than in Control.

**Figure 4:**
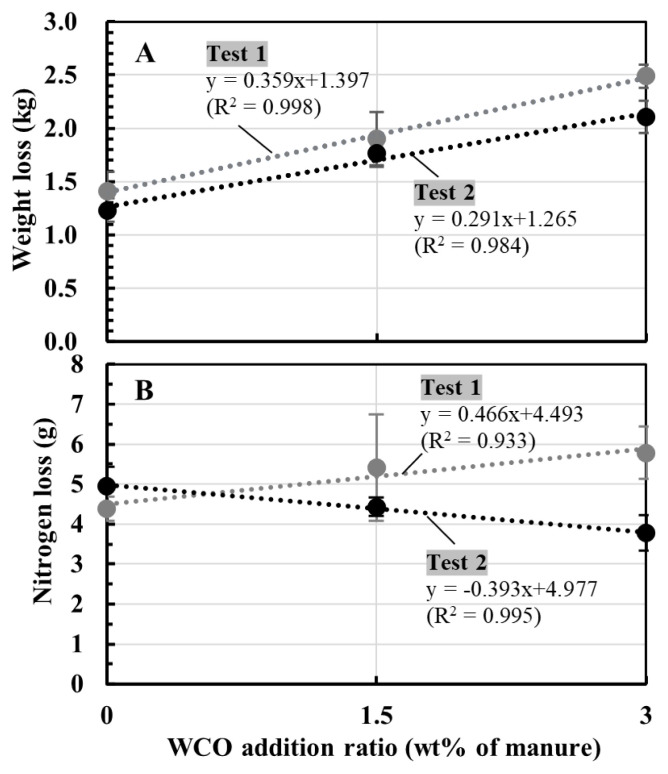
Relations between the WCO addition ratio and the losses of weight and nitrogen of the composted mixtures in Tests 1 and 2. Plotted values of the losses are the average values, and bars on the symbols are standard deviations (shown in Tables 2 and 3).

Figure 4 shows the correlations between the WCO addition ratio (0 to 3 wt% of the manure) and weight and nitrogen losses of composted mixture during composting in Tests 1 and 2. Between the addition ratio and weight loss, positive linear correlations were observed in both tests (Figure 4A). On the other hand, a positive linear correlation between the WCO content and the nitrogen losses in Test 1 and a negative linear correlation between the two parameters in Test 2 (Figure 4B).

## DISCUSSION

WCO is both an organic waste product and a recycling resource. In Japan, the annual WCO production in 2017 was 520,000 to 540,000 tons, of which approximately 390,000 to 430,000 tons were recycled to create various products, including livestock feed, ink, paint, soap, and fuel; however, the remainder WCO was incinerated or disposed in landfills [22,23]. Utilization in the composting treatment of animal manure might promote recycle use of WCO.

In both tests in this study (Tests 1 and 2), significant reductions in the total weight and water content of the composted mixtures were observed with increasing WCO contents (Tables 2, 3; Figure 4A). Several studies have investigated the effects of adding WCO or waste white clay, both of which are characterized by high lipid contents, on the composting of organic wastes; subsequently, accelerated drying and reduction of total weight of composted materials were observed [24–26]. These observations indicated that WCO addition promoted weight reduction of the composted material during composting under the wide range of aeration condition.

Conversely, the NH_3_ emissions and nitrogen loss trends observed during composting in Tests 1 and 2 differed from each other. In both tests, the collected NH_4_-N accounted for approximately 90% of the total nitrogen losses for all samples, with some unavoidable nitrogen losses observed during the turnings (Tables 2, 3). Hence, NH_3_ emissions were responsible for most of the nitrogen losses during composting. In Test 1, NH_3_ concentrations within the first 7 d of composting decreased marginally in WCO1.5 and WCO3 treatments compared with those in Control (Figure 2B); however, the collected NH_4_-N and nitrogen losses increased with increasing WCO contents (Table 2; Figure 4B). The inconsistency between these results could be because of deviations between the measured and actual NH_3_ concentrations that occurred probably due to intermittent measurements. Moreover, the dissolution of larger amounts of emitted NH_3_ in the larger condensed water in the WCO-added treatments than in Control, prior to measurement at the gas sampling port of the experimental system (Figure 1), may have caused the inconsistencies. Conversely, in Test 2, NH_3_ concentrations decreased within the first 7 d of composting in the WCO-added treatments (Figure 3B). Moreover, nitrogen losses and collected NH_4_-N amount decreased as the WCO content increased, and significant differences (p<0.05) were observed in the two parameters between WCO3 and Control samples (Table 3; Figure 4B). These results indicated that aeration in the composting mixtures influenced the impact of adding WCO on NH_3_ emissions during composting.

The addition of WCO in composting was expected to influence NH_3_ emissions owing to its characteristics or metabolic reaction of microorganisms. In both composting tests, organic nitrogen contents increased in all three treatments although the VS and total nitrogen contents decreased (Tables 2, 3) by the end of composting. The increase in organic nitrogen content was proportional to the WCO content and was more evident in the WCO-added treatments in Test 2 than in Test 1. During composting, microorganisms must have generated NH_4_-N during the decomposition of organic matter, while simultaneously assimilating NH_4_-N during the synthesis of new microbial cells, and used the added WCO for both reactions. In this study, the balances of these reactions might have differed between WCO-added treatments in Test 1 and Test 2, causing differences in the NH_3_ emissions between the tests. On the other hand, the addition of WCO decreased the pH of the initial mixtures (Tables 2, 3), and increased temperatures in WCO1.5 and WCO3 treatments (Figures 2A, 3A). The decrease in the pH might have restricted NH_3_ emissions in the early stage of composting, whereas the increased temperatures might have accelerated the emissions. However, these phenomena occurred in both Tests 1 and 2. The pH increased to 8.3 to 8.7 in all samples by the end of composting, and the NH_4_-N contents in the final mixtures were less in WCO1.5 and WCO3 treatments than in Control. These observations suggested that pH decrease and heat generation caused by the addition of WCO did not substantially contribute to the different NH_3_ emission trends observed in Tests 1 and 2.

Previous studies on the effect of CO or WCO during composting reported reduced NH_3_ emissions even under high aeration rates (≥35 L/min/m^3^ of the composting material) [12,13,15]. In these studies, the contents of CO or WCO (≥10 wt% of the composting material) exceeded the amounts used in this study (≤3 wt% of the manure). Conversely, Furuya et al [14] reported a significant reduction in NH_3_ emissions with the addition of CO in the composting of swine manure without forced aeration. In this report, the CO addition ratio was set to 3.7 wt% of the composting mixture of manure and sawdust, close to the WCO addition ratio in the present study. To obtain reduction of NH_3_ emissions by adding WCO at the addition ratio ≤3–4 wt% of the manure, aeration condition during composting should be considered, and composting under low aeration rate or without forced aeration might be effective.

Future studies can focus on assessing the effect of adding WCO in reducing NH_3_ emissions in the settings of WCO addition ratio and aeration rate in this study (≤3 wt% of the manure and ≈23 L/min/m^3^ of the material) in large-scale composting treatments in reducing NH_3_ emissions to explore the potential applications of this method. Additionally, the impact of WCO addition on the quality of the prepared compost should be examined.

## Figures and Tables

**Table 1:** Composting test parameters (Test 1 and Test 2)

Tests	Treatments	Materials (kg)			Initial mixtures (kg)^2)^

		Manure^1)^	Sawdust^1)^	WCO	
Test 1	Control	9.00	1.80	-	10.00
	WCO1.5	9.00	1.80	0.135 (1.5)^3)^	10.125
	WCO3	9.00	1.80	0.270 (3.0)	10.250
Test 2	Control	9.30	1.55	-	10.00
	WCO1.5	9.30	1.55	0.140 (1.5)	10.129
	WCO3	9.30	1.55	0.279 (3.0)	10.257

WCO, waste cooking oil.

1) The weight ratios of manure and sawdust (manure/sawdust) differed marginally in the two tests: 5/1 in Test 1 and 6/1 in Test 2.

2) The values in this column indicate the initial weights of the composting mixtures placed in the composting apparatuses. Considering added amounts of WCO, the weights in WCO1.5 and WCO3 treatments were higher than that in Control.

3) The values in parentheses are weight % of WCO to dairy manure.

* Aeration rate (L/min) in Test 1 was changed during the composting period; 0.55 (day 0 to 7), 0.50 (day 7 to 14), and 0.45 (day 14 to 20), and it was fixed at 0.3 in Test 2.

**Table 2:** Changes in the contents and pH of the composting mixtures in Test 1

Items	Initial mixture			Final mixture		
	
	Control	WCO1.5	WCO3	Control	WCO1.5	WCO3
pH	7.34±0.26	7.18±0.09	7.07±0.07	8.72±0.26	8.42±0.41	8.33±0.44
Total weight (kg)	10.000	10.125	10.250	8.59±0.18^a^	8.22±0.25^ab^	7.76±0.11^b^
Water	7.32±0.09	7.36±0.11	7.39±0.14	6.44±0.26^a^	5.98±0.27^b^	5.49±0.20^c^
VS	2.48±0.11	2.58±0.11	2.68±0.13	1.95±0.10	2.04±0.06	2.07±0.15
Ash	0.20±0.02	0.19±0.01	0.18±0.01	0.20±0.02	0.20±0.02	0.20±0.02
Weight loss (kg)	-	-	-	1.41±0.18^a^	1.91±0.25^b^	2.49±0.11^c^
Condensed water^1)^	-	-	-	0.86±0.15^a^	1.28±0.19^b^	1.67±0.15^b^
Drain water^2)^	-	-	-	0.05±0.01	0.04±0.01	0.06±0.02
Loss at turnings^3)^	-	-	-	0.08±0.03	0.08±0.02	0.10±0.03
Other loss	-	-	-	0.42±0.07^a^	0.50±0.09^ab^	0.67±0.09^b^
Total nitrogen (g)	40.56±0.98	40.57±1.51	40.62±0.88	36.17±0.98	35.16±0.18	34.84±0.59
Organic nitrogen	29.05±1.32	28.47±1.39	28.26±0.95	31.79±1.66	31.69±1.63	31.99±0.79
NH_4_-N	11.45±1.32	11.91±0.75	12.30±1.35	4.35±2.36	3.44±1.81	2.49±0.65
NO_x_-N	0.06±0.06	0.19±0.17	0.06±0.10	0.01±0.01	0.03±0.05	0.02±0.04
Nitrogen loss (g)	-	-	-	4.39±0.30	5.41±1.33	5.78±0.65
Collected NH_4_-N^4)^	-	-	-	3.94±0.21	4.88±1.30	5.10±0.62
Other loss	-	-	-	0.45±0.08^a^	0.53±0.06^ab^	0.69±0.05^b^

Data are shown as means±standard daviations of the repeated composting test (n = 3).

VS, volatile solids; NH_4_-N, ammonium nitrogen; NO_x_-N, nitrous or nitric nitrogen.

1) Total value of condensed water collected from the inside and outside the composting apparatus.

2) Total value of drain water collected from inside the vessel.

3) Total value of weight losses at turnings.

4) Total value of NH_4_-N in the liquid samples (condensed water, drain water, and NH_3_-collecting liquid).

a–c The different characters following the values indicate significant differences (p<0.05) between the values in the same issues.

**Table 3:** Changes in the contents and pH of the composting mixtures in Test 2

Items	Initial mixture			Final mixture		
	
	Control	WCO1.5	WCO3	Control	WCO1.5	WCO3
pH	7.57±0.09	7.41±0.21	7.07±0.26	8.63±0.22	8.66±0.17	8.67±0.17
Total weight (kg)	10.000	10.129	10.257	8.77±0.11^a^	8.36±0.13^ab^	8.15±0.15^b^
Water	7.40±0.12	7.42±0.17	7.43±0.17	6.65±0.06^a^	6.27±0.13^b^	6.00±0.08^c^
VS	2.38±0.12	2.51±1.25	2.62±0.17	1.90±0.10	1.89±0.02	1.97±0.09
Ash	0.22±0.02	0.20±0.05	0.21±0.04	0.22±0.03	0.21±0.02	0.22±0.03
Weight loss (kg)	-	-	-	1.23±0.11^a^	1.77±0.13^b^	2.11±0.15^c^
Condensed water^1)^	-	-	-	0.77±0.07^a^	1.27±0.04^b^	1.42±0.32^b^
Drain water^2)^	-	-	-	0.06±0.06	0.10±0.09	0.16±0.16
Loss at turnings^3)^	-	-	-	0.09±0.01	0.12±0.03	0.15±0.03
Other loss	-	-	-	0.42±0.07	0.50±0.09	0.67±0.09
Total nitrogen (g)	40.40±0.68	40.24±0.88	40.18±0.78	35.44±0.53	35.81±0.80	36.41±1.13
Organic nitrogen	27.56±1.11	26.64±0.23	26.77±0.64	29.94±1.15	31.03±0.38	31.88±0.79
NH_4_-N	12.77±0.75	13.59±1.04	13.41±0.75	5.47±1.04	4.77±1.16	4.52±1.10
NO_x_-N	0.07±0.09	0.02±0.03	0.01±0.02	0.03±0.05	0.01±0.02	0.01±0.03
Nitrogen loss (g)	-	-	-	4.95±0.48^a^	4.43±0.24^ab^	3.78±0.44^b^
Collected NH_4_-N^4)^	-	-	-	4.44±0.39^a^	3.96±0.18^ab^	3.44±0.48^b^
Other loss	-	-	-	0.51±0.10	0.47±0.07	0.34±0.12

Data are shown as means±standard deviations of the repeated composting test (n = 3).

VS, volatile solids; NH_4_-N, ammonium nitrogen; NO_x_-N, nitrous or nitric nitrogen.

1) Total value of condensed water collected from the inside and outside the composting apparatus.

2) Total value of drain water collected from inside the vessel.

3) Total value of weight losses at turnings.

4) Total value of NH_4_-N in the liquid samples (condensed water, drain water, and NH_3_-collecting liquid).

a–c The different characters following the values indicate significant differences (p<0.05) between the values in the same issues.
